# The Analysis of A Frequent *TMPRSS3* Allele Containing P.V116M and P.V291L in A Cis Configuration among Deaf Koreans

**DOI:** 10.3390/ijms18112246

**Published:** 2017-10-26

**Authors:** Ah Reum Kim, Juyong Chung, Nayoung K. D. Kim, Chung Lee, Woong-Yang Park, Doo-Yi Oh, Byung Yoon Choi

**Affiliations:** 1 Samsung Genome Institute, Samsung Medical Center, Seoul 06351, Korea; amorkimar@naver.com (A.R.K.); bionkdk@gmail.com (N.K.D.K.); spinelyc@gmail.com (C.L.); woongyang@gmail.com (W.-Y.P.); 2 Department of Otorhinolaryngology-Head and Neck Surgery, Wonkwang University College of Medicine, Iksan 54538, Korea; claudia7974@hanmail.net; 3 Department of Molecular Cell Biology, School of Medicine, Sungkyunkwan University, Seoul 06351, Korea; 4 Department of Otorhinolaryngology-Head and Neck Surgery, Seoul National University Bundang Hospital, Seoul National University College of Medicine, Seongnam 13620, Korea; dooyi9@gmail.com; 5 Sensory Organ Research Institute, Seoul National University Medical Research Center, Seoul 03080, Korea

**Keywords:** deafness, *TMPRSS3* mutation, DFNB8/10, cochlear implantation, sensorineural hearing loss

## Abstract

We performed targeted re-sequencing to identify the genetic etiology of early-onset postlingual deafness and encountered a frequent *TMPRSS3* allele harboring two variants in a cis configuration. We aimed to evaluate the pathogenicity of the allele. Among 88 cochlear implantees with autosomal recessive non-syndromic hearing loss, subjects with *GJB2* and *SLC26A4* mutations were excluded. Thirty-one probands manifesting early-onset postlingual deafness were sorted. Through targeted re-sequencing, we detected two families with a *TMPRSS3* mutant allele containing p.V116M and p.V291L in a cis configuration, p.[p.V116M; p.V291L]. A minor allele frequency was calculated and proteolytic activity was measured. A p.[p.V116M; p.V291L] allele demonstrated a significantly higher frequency compared to normal controls and merited attention due to its high frequency (4.84%, 3/62). The first family showed a novel deleterious splice site variant—c.783-1G>A—in a trans allele, while the other showed homozygosity. The progression to deafness was noted within the first decade, suggesting DFNB10. The proteolytic activity was significantly reduced, confirming the severe pathogenicity. This frequent mutant allele significantly contributes to early-onset postlingual deafness in Koreans. For clinical implication and proper auditory rehabilitation, it is important to pay attention to this allele with a severe pathogenic potential.

## 1. Introduction

Hearing loss is one of the most common diseases in newborns [1]. It is estimated that in all reported cases of genetic hearing loss, syndromic hearing loss accounts for approximately 30% and non-syndromic sensorineural hearing loss (SNHL) for about 70% [2]. To date, at least 159 genetic loci have been mapped for non-syndromic SNHL (http://hereditaryhearingloss.org). Among the 67 genes mapped for non-syndromic autosomal recessive hearing loss, *TMPRSS3* (MIM# 601072, NM_024022) has been determined to be a causative gene for autosomal recessive (DFNB8/10) SNHL [3].

*TMPRSS3* encodes a transmembrane serine protease that is composed of 454 amino acids [4]. *TMPRSS3* includes 13 exons and is located on chromosome 21q22.3 [5]. Interestingly, mutations in this gene have been shown to be related to two discrete auditory phenotypes, depending on the protease activities of mutant proteins [6]. A close association has been reported between remaining protease activity and residual hearing, highlighting a genotype-phenotype relationship [7]. In detail, a combination of two “severe” *TMPRSS3* mutations with null protease activity in a trans configuration leads to profound deafness with prelingual onset (DFNB10), while the severe mutation—in combination with milder *TMPRSS3* mutations with a significant residual protease activity—leads to a milder phenotype with postlingual onset (DFNB8) [8]. Moreover, we previously showed that among those Koreans with sporadic or autosomal recessive severe SNHL with significant residual low-frequency hearing that went away mostly during early childhood and early adolescent years, 11.2% carried the variants of this gene, suggesting that DFNB8, rather than DFNB10, is a more important *TMPRSS3*-related phenotype in Koreans [8].

Here, we report a frequent *TMPRSS3* mutant allele containing p.V116M and p.V291L in a cis configuration among Koreans with a severe degree of postlingual SNHL. The first family carried a novel and likely pathogenic splice site variant in the trans allele. In the second family, the affected subject showed homozygosity for this allele. The pathogenic potential of this allele carrying two variants in a cis configuration has never been reported. Thus, we aimed to elucidate the pathogenic potential of this allele and to correlate it with an already-established relationship between genotype and phenotype.

## 2. Results

### 2.1. Clinical Phenotype

**Figure 1:**
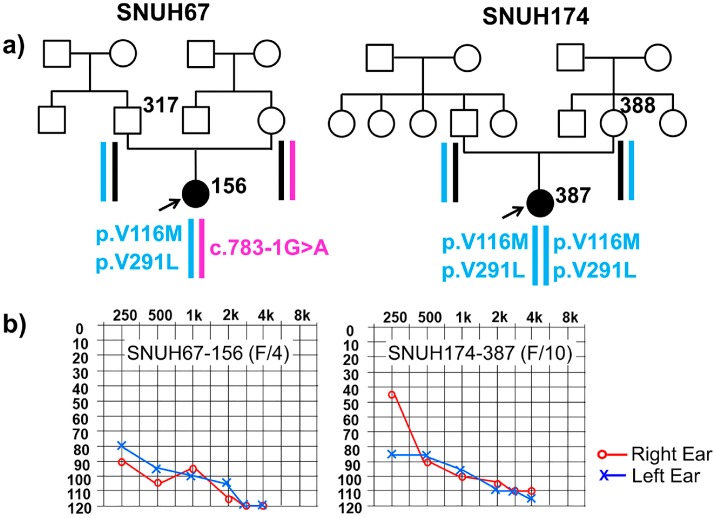
Pedigrees of the two families related to *TMPRSS3* and audiograms of affected subjects. (**a**) In this pedigree, the most likely haplotype of *TMPRSS3* is reconstructed based on the segregation study. The p.[p.V116M; p.V291L] allele (grey box) is shared by the two unrelated probands (red arrow). (**b**) Pure tone audiometry obtained from both probands directly before cochlear implantation is presented. Severe-to-profound hearing loss with minimal residual hearing is shown. Red colored graph refers to left-sided hearing loss and blue colored graph refers to right-sided hearing loss.

Pure-tone audiograms of the affected subjects from the two families carrying potentially pathogenic *TMPRSS3* variants showed bilateral, symmetrical, and severe-to-profound non-syndromic SNHL with either perilingual or postlingual childhood onset (Figure 1b). SNUH67-156 had a substantial degree of residual hearing in early childhood, according to her parents. However, she rapidly lost her hearing from the age of 3. At the age of 4, she had severe-to-profound hearing loss and underwent cochlear implantation in the same year. Her family participated in this study when she became 6 years old. Subject SNUH174-387 had significant hearing loss, which began at the age of 5 years, which progressed to severe hearing loss with little preservation of low-frequency hearing five years later. She also underwent cochlear implantation at the age of 10 years. Immediately after cochlear implantation, she was recruited for this study.

### 2.2. Variant Detection by Targeted Resequencing Data Analysis

We paid attention to two remarkable missense variants, which were shared by two independent subjects—SNUH67-156 and SNUH174-387—with clinical similarities. The targeted resequencing data from TRS-204 for SNUH67-156 and TRS-129 for SNUH174-387 were checked against the human reference genome and unrelated non-pathogenic SNPs were filtered out under an autosomal recessive inheritance pattern. Twelve and nine candidate variants, including clinically pathogenic flagged SNPs, remained from the two families (Table 1). Among these, we further excluded nine and seven variants that did not co-segregate with the SNHL, leading to an identification of variants from only one gene. These variants were c.G346A (p.V116M) in exon 5, c.G871C (p.V291L) in exon 9, and c.783-1G>A in intron 8 (Table 2). A segregation study also confirmed a phase configuration of the alleles in these two families: two variants, p.V116M and p.V291L, in one allele (p.[p.V116M; p.V291L]) and c.783-1G>A in the other allele were noted from SNUH67 and p.[p.V116M; p.V291L] was detected as a homozygote in SNUH174 (Figure 1).

### 2.3. In Silico Prediction of Pathogenic Potential

**Figure 2:**
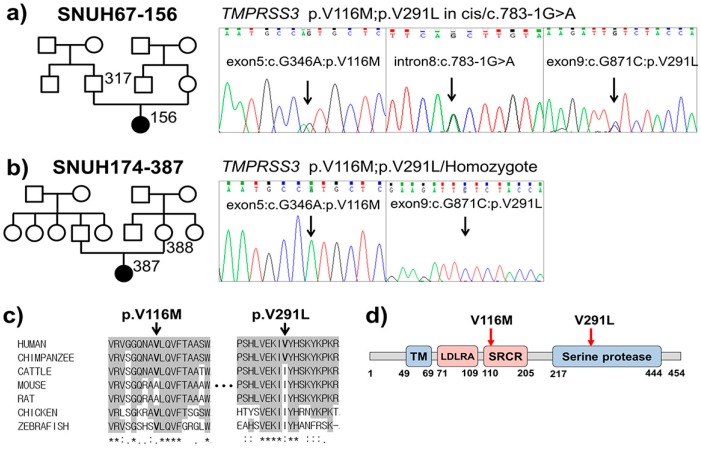
Sanger sequencing traces, conservation, and residing domains of *TMPRSS3* variants in this study. (**a**) Sanger sequencing chromatograms show p. V116M, p.V291L, and c.783-1G>A variants in a heterozygous fashion from SNUH67-156. (**b**) The variants, p. V116M and p.V291L, are in a homozygous fashion from SNUH174-387. (**c**) Conservation status of the two missense variants among various species from human to zebrafish. (**d**) Two missense variants in its domain of *TMPRSS3*; The residue of p.V116 and p.V291is located in the SRCR and the serine protease domain, respectively.

Of the two missense variants residing in the same allele in a cis configuration, p.V116M—located in the SRCR domain (Figure 2d)—was suggested to be pathogenic for the following four reasons: firstly, this variant was previously detected in an Indian family segregating an autosomal recessive SNHL; secondly, this variant was predicted to be damaging by SIFT (http://www.fruitfly.org/seq_tools/splice.html) and to be damaging by Polyphen-2 (http://genetics.bwh.harvard.edu/pph2/); thirdly, this previously reported missense variant was not detected in our 426 unrelated Korean control chromosomes, supporting its pathogenic potential; and finally, this variant was highly conserved from humans to zebrafish with high GERP++ score of 4.94. On the other hand, the other missense variant, p.V291L, in the serine protease domain (Figure 2d), was predicted to be non-pathogenic with poor conservation among species, from humans to zebrafish (Figure 2c). Additionally, another study from Korea reported that p.V291L was likely to be a rare polymorphism (Table 2) [9].

However, the pathogenic effect of a novel splicing site variant detected in *trans* to the p.[p.V116M; p.V291L] allele seemed to be undisputable. The critical effect to abolish canonical splice site was predicted in accordance with the two splice site prediction programs (scoring: from 0.94 to 0 by BDGP and from 11.22 to 0 by ESE finder). Therefore, the novel splice site variant was regarded as severely pathogenic since it was predicted to cause truncation of the protein and several variants (ex. p.Q398X, and c.1180_1187del8ins68) which truncate protein at more downstream to our splice site variant site had already been reported to be pathogenic [8].

### 2.4. Assessment of Minor Allele Frequency

We tried to predict the pathogenic effect of the p.[p.V116M; p.V291L] allele by making a comparison of the minor allele frequency (MAF) of this allele between the Korean deaf population and normal controls. The MAF of p.V116M from the four databases (UCSC genome browser, 1000G, KRGDB, and SGI Phenotype-documented in-house WES database) was reported to be 0.0015, 0, 0, and 0, respectively. The second variant, p.V291L—considered to be less pathogenic than p.V116M—was also detected with a low frequency of 0.00005, 0.0006, 0.0008, and 0 from the same four databases (Table 2). Therefore, the probability for the two variants to reside in the same allele among normal controls was estimated to be no more than 0.00005, 0, 0, and 0, based on MAF of each allele from four corresponding databases, even if two variants were tightly linked. These figures obtained from databases with either Korean normal controls (KRGDB and SGI Phenotype-documented in-house WES database) or general normal controls (UCSC genome browser and 1000G) were strikingly lower than 0.048 (3/62 alleles), which was the detection rate of the p.V116M-p.V291L allele among 31 postlingual SNHL subjects (*p* < 0.0001, Fisher’s exact test (two-tailed)). The association between the postlingual SNHL and the p.[p.V116M; p.V291L] allele was considered extremely significant.

### 2.5. In Vitro Yeast Based Protease Assay

**Figure 3:**
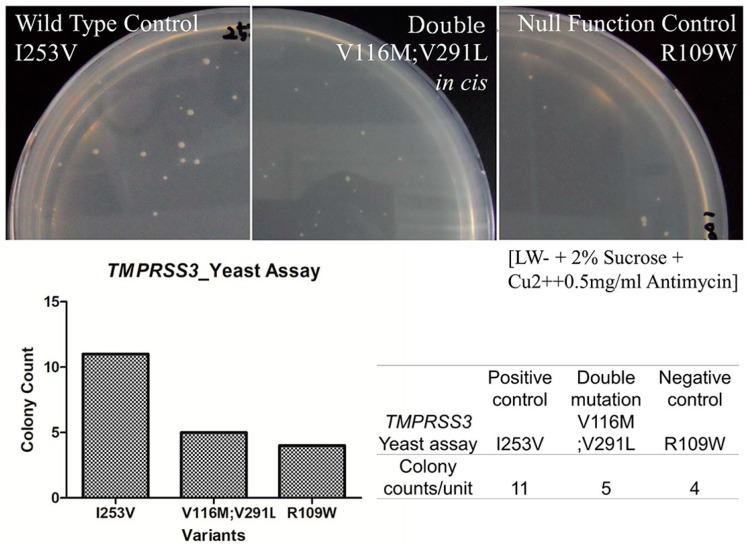
Assay for the proteolytic activity of the transformants of each genotype of *TMPRSS3*. Colonies were counted with the criteria in a well-shaped order. Far left panel indicates the wild type controls; right indicates null function controls. Compared with these, the middle one contains the proteolytic activity of double mutated constructs: the double mutants, p.V116M and p.V291L, show a significantly weaker proteolytic activity than the one on the left, suggesting pathogenicity. (A medium containing Leu^−^/Trp^−^ + 2% Sucrose + Cu^2+^ + 0.5 mg/mL Antimycin).

To better address the pathogenic potential of the p.[p.V116M; p.V291L] allele, we evaluated the proteolytic activities of the allele and compared them against those of the wild type and the known null function controls of *TMPRSS3*. The p.[p.V116M; p.V291L] allele showed a significantly reduced proteolytic activity, which was almost comparable to that of the null function control (Figure 3). The number of colonies with a measured area of 0.003 or higher was counted. The wild type control, the double mutated one, and the null function control showed 11, 5, and 4 surviving colonies, respectively. The number of surviving colonies from the double mutated allele was significantly reduced compared with that of the wild type control, nearly approaching the level of the null function control. Therefore, the p.[p.V116M;p.V291L] allele of *TMPRSS3* was considered severely pathogenic, pointing out that SNUH67-156 and SNUH174-387 carry a combination of severely pathogenic *TMPRSS3* alleles.

## 3. Discussion

We have previously shown that the transmembrane serine protease is tightly correlated with the auditory phenotype of hearing loss subjects carrying autosomal recessive mutations of *TMPRSS3* [8]. To date, most subjects carrying *TMPRSS3* mutations have been considered DFNB8, which can be characterized by substantial residual hearing in low frequencies like a ski-slope hearing loss and prominent gradual hearing loss that occurs especially in the second decade of life [13,14]. Many variants related to *TMPRSS3* mutation were reported in the deaf Korean population [8,12]. p.Ala306Thr in the serine protease of *TMPRSS3* and p.Arg109Trp in the SRCR were both classified as severe mutations [8,12]. p.Thr248Met in the SRCR was reported to be a mild mutation [8]. Compatible with a milder auditory phenotype of DFNB8, two Korean subjects with such an auditory phenotype in our previous report carried a relatively milder variant, such as p.T248M in a trans configuration with a severely pathogenic *TMPRSS3* variant [8]. In contrast, the phenotypes of SNUH67-156 and SNUH174-387 in the present study were considered more like DFNB10 rather than DFNB8, based on the severity of auditory phenotype with early onset in the first decade of their life that progressed to severe-to-profound hearing loss at various frequencies. Indeed, the p.[p.V116M;p.V291L] allele of *TMPRSS3* turned out likely to be severely pathogenic and the novel splice site variant c.783-1G>A was predicted to critically affect the canonical splicing.

We initially hypothesized that p.V116M in the SRCR domain alone or in conjunction with p.V291L in the serine protease domain would significantly destroy the function of the allele, based on the data available in the literature. According to the literature, detection frequency of pathogenic variants in the serine protease, SRCR, LDL receptor like domain and transmembrane domains of this gene has been recently reported to be 0.41(16/39), 0.33 (13/39), 0.20 (8/39), and 0.05 (2/39), respectively [15]. Although Lee et al. (2003) predicted that mutations in the SRCR and LDLRA domains affect the proper folding or assembly of the catalytic domain or alter protease substrate recognition and binding [16], however, there has not been any established domain-phenotype correlation in this gene. The p.V116M has previously been detected from an Indian deaf family segregating a severe-to-profound, prelingual, non-syndromic hearing loss in an autosomal recessive fashion [11]. In that study, this variant was detected to be in a trans configuration with a splice site variant—c.323-6G>A—that constituted a compound heterozygous state. The p.V116M variant was predicted to be damaging due to the p.V116 residue’s significant conservation among various species and its absence in the normal controls. The p.V291L variant of *TMPRSS3* was first detected in a deaf Korean family (K19) [12]. This variant was reported to be in a single heterozygous state in an autosomal recessive NSHL family. They were not able to find any additional mutation anywhere in the *TMPRSS3* gene. The prediction software also suggested that p.V291L is likely to be non-pathogenic, based on the prediction from publicly available softwares, SIFT (http://sift.jcvi.org/, pathogenicity prediction score = 0) and Polyphen-2 (http://genetics.bwh.harvard.edu/pph2/index.shtml, pathogenicity prediction score = 1). In addition, p.V291 residue was poorly conserved among species from human to zebrafish (Figure 2c). Taken together, they concluded that this variant was probably nonpathogenic. It is possible that the two variants in one allele may produce either a synergic or rescue effect on pathogenicity compared with the effect of the p.V116M variant. Indeed, there was an example of the latter case in one of the most frequent deafness genes. Using an in vitro functional analysis of the pathogenicity of four haplotypes generated by the two *GJB2* variants (p.V27I and p.E114G), the p.E114G protein showed abnormal channel activities while p.V27I showed normal channel activities [9]. Interestingly, the p.[p.V27I;p.E114G] allele were associated with less pathogenicity, suggesting that the p.V27I variant may compensate for the abnormality of its channel activities when it coexists with the p.E114G type [9]. Based on the data from the present study, the pathogenicity of the p.[p.V116M;p.V291L] allele did not seem to follow the p.[p.V27I;p.E114G] allele of *GJB2*. Using the yeast assay and ensuring visualization of the proteolytic activity, we observed that the proteolytic activity of the p.[p.V116M;p.V291L] allele of *TMPRSS3* was just as poor as that of the null function control. It is likely that p.V116M and p.V291L of *TMPRSS3* did not, at least, rescue the pathogenic effect of each other. However, the synergistic effect of p.V116M and p.V291L could not be clarified in the present study, since we did not perform an in vitro functional analysis to assess the pathogenicity of each of the two variants.

The pathogenic potential of the p.[p.V116M; p.V291L] allele of *TMPRSS3* is also supported by the significantly low MAF of p.V116M and p.V291L, based on the UCSC genome browser (https://genome.ucsc.edu/), which was 0.150% and 0.005% respectively. In addition, the MAF of p.V116M and p.V291L, based on 1000G/KRGDB, was 0%/0% and 0.06%/0.08%, respectively. If the two variants are completely unrelated then it would be extremely unlikely for these two variants to be in the same allele. Alternatively, it is also possible that the two variants in cis are tightly linked and they are in strong linkage disequilibrium. Even in this case, the p.[p.V116M; p.V291L] allele of *TMPRSS3* would still be extremely rare compared with 4.8% (3/62 alleles) among postlingual SNHL subjects, strongly suggesting its pathogenic potential. Additionally, the p.[p.V116M; p.V291L] allele may also be another common founder allele of *TMPRSS3* with a strong pathogenic potential in Koreans, just as much as the p.A306T allele. Therefore, screening for the p.[p.V116M; p.V291L] allele, in addition to the previously documented common founder allele, p.A306T, of *TMPRSS3* would be an efficient way to detect DFNB8/10 in Koreans.

Furthermore, our results again confirmed a previously proposed genotype-phenotype correlation about this gene. This correlation should be kept in mind when considering auditory rehabilitation for these DFNB8/10 subjects. For those with a combination of severely pathogenic *TMPRSS3* variants, rapid aggravation of the residual hearing should be anticipated and treated accordingly. Our confirmation of the genotype-phenotype correlation of the *TMPRSS3* gene may pave the way for the establishment of a personalized auditory rehabilitation.

## 4. Materials and Methods

### 4.1. Subjects and Clinical Findings

All procedures in this study were approved by the Institutional Review Boards of Seoul National University Hospital (IRBY-H-0905-041-281) and Seoul National University Bundang Hospital (IRB-B-1007-105-402). Written informed consent was obtained from affected and unaffected individuals. In the case of participants under the age of eighteen years, written informed consent was obtained from their parents or guardians on their behalf.

Two probands (SNUH67-156, F/6 and SNUH174-387, F/10 at ascertainment) with severe SNHL in an autosomal recessive fashion, and their family members who were willing to participate were enrolled in this study. However, a maternal DNA sample from the first family and a paternal DNA sample from the second family were not available due to their reluctance. Parents and their relatives were interviewed to infer comprehensive clinical history to rule out perinatal, ototoxic, infectious, and traumatic factors that could lead to non-genetic hearing loss. An evaluation of deafness was done by clinical examination, audiologic tests—including pure tone audiometry and auditory brainstem response—and imaging tests, such as temporal bone computed tomography (TBCT). Pure tone audiometry (PTA), with air and bone conductions at frequencies ranging between 250 and 8000 Hz, was performed according to the standard protocols. The hearing loss range was described as follows, depending on the pure-tone audiometry results: low frequency, 250–500 Hz; mid frequency, 1–2 kHz; and high frequency, 4–8 kHz. Severity of hearing loss is graded as mild (20–40 dB), moderate (41–55 dB), moderately severe (56–70 dB), severe (71–90 dB), or profound (>90 dB). The first-visit PTA was performed on two subjects (SNUH67-156, SNUH174-387), and in both of these subjects, TBCT was obtained to identify any inner ear anomalies related to hearing loss in these families.

### 4.2. Molecular Genetic Test

After having recruited our previous cohort published in 2014 regarding *TMPRSS3* [8], we collected 88 hearing-impaired patients who underwent cochlear implantation and agreed to participate in molecular genetic testing. These patients were ascertained from two tertiary referral hospitals—Seoul National University Hospital (SNUH) and Seoul National University Bundang Hospital (SNUBH)—between June 2013 and October 2014. Among these 88 patients, we focused on families that segregated postlingual-onset progressive and severe autosomal recessive non-syndromic SNHL that mandated cochlear implantation. Subjects with either *GJB2* or *SLC26A4* variants were excluded. Congenital severe-to-profound SNHL cases were not included. Subjects whose hearing loss was related to trauma or infection were also excluded. Furthermore, subjects for whom the onset of hearing loss was at or over the 6th decade were not included in the present study in order to focus more on genetic SNHL cases rather than age related ones. After all exclusions, our cohort was comprised of 31 probands manifesting early onset postlingual progressive and severe hearing loss. Either of the two deafness gene panels for targeted resequencing (TRS) was utilized for molecular genetic testing; one is TRS-204 containing 204 known deafness genes by SGI (Samsung genomic institute, http://www.samsunghospital.com/dept/main/index.do?DP_CODE=BP7) and the other is TRS-129 containing 129 known deafness genes by Otogenetics Corporation (http://www.otogenetics.com/).

Raw data from the TRS technology was mapped and filtered sequentially in accordance with the exclusion criteria and the in-house flow chart of hierarchical molecular genetic tests, as we previously reported [17,18]. The obtained reads were aligned with the UCSC hg19 reference genome (https://genome.ucsc.edu/), which is an interactive website offering access to genome sequence data from a variety of vertebrate and invertebrate species, including major model organisms, integrated with a large collection of aligned annotations, with narrowed down variants. In brief, the data were filtered to select candidate variants in autosomal recessive genes regarding non-syndromic SNHL through the following bioinformatics analysis. In a basic filtering step, non-synonymous Single Nucleotide Polymorphisms (SNPs) with Q call >10 and read depths >20 were selected; the selected SNPs were compared and tagged using the Single Nucleotide Polymorphism database (dbSNP build 138) and an in-house database. In the end, only novel SNPs and SNPs causing known diseases remained. Next, we checked the inheritance patterns in each family, and excluded variants that did not co-segregate with the SNHL phenotype. More specifically, we first excluded variants that did not fit for the autosomal recessive inheritance pattern. For example, detection of only one novel variant with unknown pathogenic potential in a known recessive deafness gene indicates that the variant is likely to be a fortuitously detected SNP.

Next, we excluded variants of the autosomal dominant gene that were shared by the proband and one of the parents with normal hearing from the two families. Finally, the remaining SNPs were validated by Sanger sequencing (Figure 2a,b). The SNPs were also checked against an additional 426 unrelated Korean control chromosomes. Pathogenicity of the splicing variant was predicted using ESE finder (http://krainer01.cshl.edu/cgi-bin/tools/ESE3/esefinder.cgi?process=home) and BDGP (Berkeley Drosophila Genome Project; http://www.fruitfly.org/seq_tools/splice.html). Missense variants were predicted using SIFT (http://sift.jcvi.org/) and Polyphen-2 (http://genetics.bwh.harvard.edu/pph2/). For estimation of the evolutionary conservation of the amino acid sequences, we referred to the GERP++ score from UCSC Genome Browser (http://genome.ucsc.edu/). These filtering processes and subsequent segregation studies led us to detect two kinds of interesting *TMPRSS3* mutant alleles—one is the allele with two equal candidate variants in a cis configuration and the other is a novel splice site variant of *TMPRSS3*.

### 4.3. Prediction of Pathogenic Potential Based on the Minor Allele Frequency of the Candidate Allele of TMPRSS3

For the specific allele in *TMPRSS3* with two potentially pathogenic variants in a cis configuration, the minor allele frequency (MAF) was verified using the 1000 Genomes project data (http://www.ncbi.nlm.nih.gov/variation/tools/1000genomes/), which is a deep catalog of human genetic variation representing more than 80M short variants with genotypes for 2504 individuals across 26 populations, and KRGDB (http://152.99.75.168/KRGDB/menuPages/intro.jsp), which provides a comprehensive map of the Korean genomic variants for future disease association studies and population genetics. The MAF of the allele, including p.V116M and p.V291L in cis as well as the alleles with each of the two variants, was calculated and compared with a frequency of the alleles among Korean postlingual SNHL.

### 4.4. Yeast Based Protease Assay

To confirm the pathogenicity of the allele, we constructed expression vectors for double variants that was mutagenized from the wild type *TMPRSS3* protease vector, as previously described [16,18]. The vectors expressing the mutant protease with p.V116M and p.V291L was generated by PCR based an in vitro mutagenesis. Primer sets containing the altered nucleotide were as follows: p.V116M (5′-TGGGTGGTCAGAATGCCATGCTCCAGGTGTTCACA-3′ and 5′-TGTGAACACCTGGAGCATGGCATTCTGACCACCCA-3′) and p.V291L (5′-ACTTGGTGGAGAAGATTCTCTACCACAGCAAGTAC-3′ and 5′-GTACTTGCTGTGGTAGAGAATCTTCTCCACCAAGT-3′).

Proteolytic activities of *TMPRSS3* and its variants were assayed, as described previously [16,19].

Briefly, a yeast strain KSY01 (MATa, leu2 ura3 his3 trp1 lys2 suc2-D9 kex2::HIS3) was co-transformed with the wild type and mutant *TMPRSS3* expression vectors, as well as the substrate vector. The Leu+/Trp+ transformants were selected on minimal media containing 2% glucose without Leu and Trp, and then a replica was plated on YPD media containing 2% sucrose and 0.5 mg/mL antimycin A. Colonies usually appeared within seven days at 30 °C. Quantification of the number of colonies in the plates was performed using ImageJ program (https://imagej.net/). We selected only colonies with a measured area of 0.003 or higher.

## Figures and Tables

**Table 1:** List of the variants surviving from initial filtering based on the TRS200, TRS129 analysis. Details of final candidates after targeted re-sequencing of the 200, 129 deafness genes, respectively.

	Gene	Exon	Nucleotide Change	Amino Acid Change	Pathogenicity Prediction	Zygosity
					SIFT	
SNUH67-156	*HSPG2*	exon85	c.G11602A	p.V3868M	Tolerated	Hetero
	*KCNQ4*	exon1	c.T140C	p.L47P	Tolerated	Hetero
	*TMIE*	exon4	c.391_393delAAG	p.K131del	NA	Hetero
	*GPR98*	exon9	c.A1567G	p.M523V	Tolerated	Hetero
	*LAMA2*	exon33	c.A4772G	p.Q1591R	Tolerated	Hetero
	*HOXA1*	exon1	c.215_223delATCACCACC	p.H72_H74del	NA	Hetero
	*TNC*	exon3	c.G1823A	p.R608H	Tolerated	Hetero
	*POU4F1*	exon2	c.486_487insGGC	p.P163delinsGP	NA	Hetero
	TMPRSS3*	exon9	c.G871C	p.V291L	NA	Hetero
	TMPRSS3*	intron9	c.783-1G>A	NA	NA	Hetero
	TMPRSS3*	exon5	c.G346A	p.V116M	NA	Hetero
	*TRIOBP*	exon7	c.1613_1615delTGT	p.L538_S539delinsP	NA	Hetero
SNUH174-387	*COL9A2*	exon30	c.G1741A	p.V581I	Tolerated	Hetero
	*FGFR3*	exon3	c.G193A	p.G65R	Tolerated	Hetero
	*COL11A2*	exon5	c.G688T	p.G230W	Damaging	Hetero
	*TPRN*	exon1	c.C761T	p.S254L	Tolerated	Hetero
	*CDH23*	exon40	c.G5411A	p.R1804Q	Tolerated	Hetero
	*KCNQ1*	exon16	c.G1927A	p.G643S	Tolerated	Hetero
	*MYO1C*	exon28	c.G2785A	p.D929N	Tolerated	Hetero
	TMPRSS3*	exon9	c.G871C	p.V291L	NA	Homo
	TMPRSS3*	exon5	c.G346A	p.V116M	NA	Homo

* Causal variants based on the autosomal recessive inheritance pattern in two probands, respectively.

**Table 2:** Overview of the three variants in *TMPRSS3*.

Exon/Intron	Nucleotide Change	Amino Acid Change	Domain	Minor Allele Frequency				Phenotype	References
				UCSC	1000G	KRGDB	SGI		
Exon5	c.G346A	p.V116M	SRCR	0.0015	0	0	0	^†^ Profound	[10,11]
Intron8	c.783-1G>A	-	-	0	0	0	0	Likely pathogenic	This Study
Exon9	c.G871C	p.V291L	Serine Protease	0.00005	0.0006	0.0008	0	Uncertain	[12]

UCSC (University of California, Santa Cruz) genome browser, GRCh37 hg19: a database of genomic sequence and annotation data for a wide variety of organisms; 1000G (Genomes Project data): extensive genome annotation, such as protein-coding genes and whole-genome regulatory information though the dedicated 1000 Genomes browser; KRGDB: Total 622 individuals from the Korean Reference Genome Database; SGI (Samsung genomic institute) in house database Korean 1000; ^†^Profound, Childhood onset (10–12 years) affecting all frequencies or prelingual profound deafness.
